# Maternal organic selenium supplementation during gestation enhances muscle fiber area and muscle fiber maturation of offspring in porcine model

**DOI:** 10.1186/s40104-022-00773-5

**Published:** 2022-11-04

**Authors:** Yan Lin, Hui Yan, Lei Cao, Daolin Mou, Dajiang Ding, Binting Qin, Lianqiang Che, Zhengfeng Fang, Shengyu Xu, Yong Zhuo, Jian Li, Jianping Wang, Chao Huang, Yuanfeng Zou, Lixia Li, De Wu, Bin Feng

**Affiliations:** 1 Animal Nutrition Institute, Sichuan Agricultural University, Chengdu, China; 2 Key Laboratory of Animal Disease-Resistant Nutrition of Ministry of Education, Sichuan Agricultural University, Chengdu, China; 3 College of Veterinary Medicine, Sichuan Agricultural University, Chengdu, 611130 Sichuan China

**Keywords:** Antioxidation, HMSeBA, Muscle, Offspring, Sows

## Abstract

**Background:**

Organic selenium supplementation during gestation improves the antioxidant status and reproductive performance of sows and increases the antioxidative capacity of the intestines of their offspring. This study was conducted to investigate the effect of maternal basel diet (control) supplemented with an organic Se, 2-hydroxy-4-methylselenobutanoic acid (HMSeBA), or inorganic sodium selenite (Na_2_SeO_3_) during gestation on the antioxidant status and development of muscle in newborn and weaned piglets. Newborn piglets before colostrum intake and weaned piglets were selected for *longissimus dorsi* (LD) muscle collection and analysis.

**Results:**

The results showed that maternal HMSeBA supplementation increased the muscle area and content of Se in the LD muscle of newborn piglets, improved gene expression of selenoproteins, and decreased oxidative status in the LD muscle of both newborn and weaned piglets compared with the control. The expression of muscle development-related genes of newborn piglets in the HMSeBA group was lower than in the control group, whereas the expression of *MRF4* in weaned piglets was higher in the HMSeBA group than in the control and Na_2_SeO_3_ groups. In addition, HMSeBA supplementation decreased the mRNA expressions of myosin heavy chains *(MyHC) IIx* and *MyHC IIb* and the percentage of *MyHC IIb*; increased the expression of *PGC-1α* in the LD muscle of newborn piglets; increased the gene expression of *MyHC IIa*; and decreased the protein expression of slow MyHC and the activity of malate dehydrogenase in the LD muscle of weaned piglets compared with the control group.

**Conclusions:**

Maternal HMSeBA supplementation during gestation can improve the antioxidative capacity of the muscle of their offspring and promote the maturity of muscle fibres in weaned offspring.

## Background

Meat quality is mainly determined by the type of muscle fibre and is positively correlated with the oxidative capacity of muscle fibres [1, 2]. Dietary supplementation with organic Se can significantly increase Se deposition in the muscles [3, 4], which in turn increases muscle antioxidant levels [3]. Several reports have shown that feeding with organic Se improves the amount of Se transferred from sows to their progeny [5, 6], thereby enhancing the antioxidative capacity of their offspring [7]. Our previous studies found that maternal supplementation with 2-hydroxy-4-methylselenobutanoic acid (HMSeBA) during gestation increased the plasma concentration of total Se and improved the antioxidative capacities of sows and their offspring [8]. However, little is known about the effects of Se source on muscle development in offspring.

Skeletal muscle accounts for 40%–50% of body weight [9]. Muscle development is regulated by several transcription factors. The MyoD family of myogenic regulatory factors (MRFs) are master regulators of myogenic determination and differentiation, while postpartum satellite cells are determined by paired box gene 7 (*Pax7*) [10]. In addition, myostatin (MSTN) inhibits muscle development [11], while mammalian target of rapamycin (mTOR) promotes muscle hypertrophy [12]. Insulin-like growth factors promote myogenesis and postnatal muscle growth by accelerating protein synthesis and inhibiting protein degradation [13]. Further, Se is involved in the differentiation of chicken embryonic myoblasts [14] and improves the fatty acid composition in poultry muscle tissues [15]. Although Se has an effect on muscle pH and drip loss in growing pigs, the effect of maternal Se supply on offspring muscle development is not clear and is worth exploring.

In most mammals, the number of muscle fibres is determined at birth. Thus, the increase in postnatal skeletal muscle mass results from an increase in muscle fibre size (hypertrophy) [16]. However, the composition of muscle fibre type is continuously changing postnatally [17], wherein days 1 to 14 are the critical periods for transformation. Based on the characteristics of its contraction, skeletal muscle fibre types are classified as slow-twitch (Type I) fibers with MyHC I expression and fast-twitch (Type II) fibers. Fast fibres are divided into type IIa (fast-twitch oxidative type) with MyHC IIa expression, type IIx (fast-twitch oxidative-glycolytic type) with MyHC IIx expression, and type IIb (fast-twitch glycolytic type) with MyHC IIb expression [18]. Muscle fibre types can transform between types I and II. Therefore, the objective of this study was to explore whether maternal addition of HMSeBA during gestation could improve muscle development, selenium deposition, and antioxidant status in the muscles of their offspring.

## Material and methods

### Experimental design and animal management

Forty-five Landrace Yorkshire sows after insemination were randomly divided into three groups according to their body weight (239.25 ± 8.54 kg) and backfat thickness (13.90 ± 1.28 mm), and received one of the following diets during gestation: basal diet (Control, *n* = 15), a basal diet supplemented with sodium selenite (Na_2_SeO_3_) at 0.3 mg Se per kg (Na_2_SeO_3_, *n* = 15), and HMSeBA at 0.3 mg Se per kg (HMSeBA, *n* = 15). The experimental diets were formulated to meet the nutrient requirements of gestating sows as recommended by NRC [19] (Table 1), except for that of selenium. All sows were fed the same lactation diet. HMSeBA (hydroxy-analogue of selenomethionine, Selisseo®, 2% Se) was provided by Adisseo France S.A.S. and Na_2_SeO_3_ (1% Se) was obtained from Chengdu Shuxing Feed Co. Ltd (Chengdu, Sichuan, China).

**Table 1:** Composition and nutrient levels of the basal diet (as-fed basis)

Item	Gestation	Lactation
Ingredients, %		
Corn	63.53	62.89
Soybean meal	14.50	22.13
Soybean oil		2.00
Wheat bran	18.00	6.00
Fish meal		2.60
_L_-Lysine HCl (98%)	0.05	0.27
_D, L_- Methionine (99%)	0.02	0.13
_L_-Threonine (98.5%)	0.05	
Limestone	1.15	0.98
Dicalcium phosphate	1.65	1.50
Choline chloride (50%)	0.15	0.15
Sodium chloride	0.40	0.40
Sodium bicarbonate		0.40
Vitamin and mineral premix	0.50^a^	0.55^b^
Total	100.00	100.00
Nutrient level^c^		
Digestible energy, Mcal/kg	3.04	3.27
Crude protein, %	14.03	17.50
Standard ideal digestible-Lysine, %	0.56	0.98
Total calcium, %	0.88	0.90
Total phosphorus, %	0.71	0.70

^a^Vitamin and mineral mixture for gestation sows supplied the following amounts of vitamins/kg and minerals/kg of complete diet: 6000 IU vitamin A; 1500 IU vitamin D_3_; 80 IU vitamin E; 2.6 mg vitamin B_1_; 6.5 mg vitamin B_2_; 3.9 mg vitamin B_6_; 15 μg vitamin B_12_; 26 mg niacin; 1.3 mg folate; 120 mg iron; 20 mg copper; 120 mg zinc; 30 mg manganese; 0.3 mg iodine. Control, 0 mg selenium/kg (analysed value is 0.13 mg selenium/kg); Na_2_SeO_3_, 0.3 mg selenium/kg (analysed value is 0.41 mg selenium/kg); HMSeBA, 0.30 mg selenium/kg (analysed value is 0.46 mg selenium/kg)

^b^Vitamin and Mineral mixture for lactation sows supplied the following amounts of vitamins/kg and minerals/kg of complete diet: 6000 IU vitamin A; 1200 IU vitamin D_3_; 50 IU vitamin E; 1.0 mg vitamin B_1_; 3.6 mg vitamin B_2_; 1.8 mg vitamin B_6_; 12.5 μg vitamin B_12_; 20 mg niacin; 12.5 mg pantothenic acid; 2.0 mg folacin; 120 mg iron; 20 mg copper; 120 mg zinc; 30 mg manganese; 0.3 mg selenium; 0.3 mg iodine

^c^Calculated value

### Sample collection

On the day of birth, 10 piglets from each group (male) were anaesthetised and sacrificed before suckling. Samples of the *longissimus dorsi* (LD) muscle from the eighth to tenth rib were collected. The remaining piglets were breastfed until they were weaned. On the day of weaning, six piglets from each group (male) were slaughtered and the LD muscle was collected and stored at –80 °C.

### Muscle fibre histological analysis

Muscle fibre morphology in pigs was determined by staining the muscle fibres using the classical ATPase method of Guth et al. [20]. All sections were photographed using a digital microscope (Nikon) based on five consecutive random areas. At least 150 muscle fibers were randomly selected by Image-Pro Plus 6.0 Image analysis software (Media Cybernetics Inc., Bethesda, MD, USA), and the diameter and area of muscle fibers in the collected images were measured [21]. The number and cross-sectional area of the muscle fibres were calculated using the software programme Image-Plus 6.0, and muscle density was calculated based on the number of muscle fibres/muscle areas.

### Measurement of selenium concentration

The Se level in the muscle was analysed according to the method of Chao et al. [3]. Briefly, approximately 0.5 g of muscle sample was digested with 10 mL HNO_3_ and 2 mL H_2_O_2_ in a microwave. The solution was then heated and treated with 6 mol/L HCl. A reagent blank test was simultaneously performed. The total Se content was determined using hydride atomic fluorescence spectrometry (AFS-9230, Beijing Auspicious Day Instrument Co., LTD, Beijing, China) [3].

### Gene expression and muscle type

Muscle tissue powder was homogenised in TRIzol reagent (Invitrogen, Shanghai, China), then RNA was extracted according to the manufacturer’s instructions and RNA concentration was determined. The expression changes of genes were validated by a SYBR-based High-Specificity miRNA qRT-PCR Detection kit (TaKaRa Biotechnology Co., Ltd., Dalian, China) on the Applied Biosystems 7900HT Real-Time PCR Detection System (Applied Biosystems, Carlsbad, USA). Real-time PCR data were analysed using the 2^-^^∆∆Ct^ method, with *GAPDH* as the reference. The primer sequences are listed in Table 2.

**Table 2:** Primer sequences for the target and reference genes

Genes	Primer	Sequence (5’ to 3’)	Accession no.
*MYHCI*	Forward	GTTTGCCAACTATGCTGGGG	AB053226.1
	Reverse	TGTGCAGAGCTGACACAGTC	
*MYHCIIa*	Forward	CTCTGAGTTCAGCAGCCATGA	AB025260.1
	Reverse	GATGTCTTGGCATCAAAGGGC	
*MYHCIIx*	Forward	TTGACTGGGCTGCCATCAAT	AB025262.1
	Reverse	GCCTCAATGCGCTCCTTTTC	
*MYHCIIb*	Forward	GAGGTACATCTAGTGCCCT	AB025261.1
	Reverse	GCAGCCTCCCCAAAAATAGC	
*GPX1*	Forward	GATGCCACTGCCCTCATGA	AF532927
	Reverse	TCGAAGTTCCATGCGATGTC	
*GPX2*	Forward	AGAATGTGGCCTCGCTCTGA	DQ898282
	Reverse	GGCATTGCAGCTCGTTGAG	
*GPX3*	Forward	TGCACTGCAGGAAGAGTTTGAA	AY368622
	Reverse	CCGGTTCCTGTTTTCCAAATT	
*GPX4*	Forward	TGAGGCAAGACGGAGGTAAACT	NM_214407
	Reverse	TCCGTAAACCACACTCAGCATATC	
*SELP*	Forward	AACCAGAAGCGCCAGACACT	EF113596
	Reverse	TGCTGGCATATCTCAGTTCTCAGA	
*TXNRD1*	Forward	GATTTAACAAGCGGGTCATGGT	AF537300
	Reverse	CAACCTACATTCACACACGTTCCT	
*TXNRD2*	Forward	TCTTGAAAGGCGGAAAAGAGAT	GU181287
	Reverse	TCGGTCGCCCTCCAGTAG	
*SELW*	Forward	CACCCCTGTCTCCCTGCAT	NM_213977
	Reverse	GAGCAGGATCACCCCAAACA	
*SEPHS2*	Forward	TGGCTTGATGCACACGTTTAA	EF033624
	Reverse	TGCGAGTGTCCCAGAATGC	
*SELO*	Forward	CTTCCGACCCCAGATGGAT	AK236851
	Reverse	GGTTCGACTGTGCCAGCAT	
*SELH*	Forward	TGGTGGAGGAGCTGAAGAAGTAC	HM018602
	Reverse	CGTCATAAATGCTCCAACATCAC	
*DIO1*	Forward	CATGGCCAAGAACCCTCACT	AY533206
	Reverse	CCAGAAATACTGGGCACTGAAGA	
*DIO2*	Forward	CGCTGCATCTGGAAGAGCTT	AY533207
	Reverse	TGGAATTGGGTGCATCTTCA	
*DIO3*	Forward	TGAAGTGGAGCTCAACAGTGATG	AY533208
	Reverse	TGTCGTCAGACACGCAGATAGG	
*GAPDH*	Forward	ACACTGAGGACCAGGTTGTG	NM_001206359
	Reverse	GACGAAGTGGTCGTTGAGGG	

According to the ratio between the mRNA expression of each myosin heavy chain subtype and type IIx mRNA (referred to as 1), the proportion of each gene in the total was calculated to obtain the proportion of muscle fibre type. The proportion of *MyHC I*, *MyHC IIa*, *MyHC IIb*, and *MyHC IIx* mRNA (%) was calculated to represent the proportion of slow oxidation, fast oxidation, fast fermentation, and intermediate type muscle fibres, respectively.

### Analysis of metabolic enzyme activities

The activities of succinic dehydrogenase (SDH), malate dehydrogenase (MDH), and lactate dehydrogenase (LDH) in the LD muscle were measured using the assay kits purchased from Nanjing Jiancheng Bioengineering Institute (Nanjing, China) and the protocol followed the manufacturer's instructions.

### Analysis of antioxidant enzyme activity and malondialdehyde content

The enzyme activities of total superoxide dismutase (T-SOD), glutathione peroxidase (GSH-Px) and catalase (CAT), total antioxidant capability (T-AOC), and malondialdehyde (MDA) level in the LD muscle were determined according to the manufacturer’s instructions (Nanjing Jiancheng Bioengineering Institute, Nanjing, China).

### Western blot

Western blotting was performed as previously reported [22]. LD muscle samples were homogenised in RIPA lysis buffer (Beyotime biotechnology, Shanghai, China) containing a protease inhibitor (Roche, Shanghai, China). Proteins were separated on 10% SDS–PAGE gel and then were transferred onto a PVDF membrane (Bio-Rad, Shanghai, China). The membrane was blocked with 5% skimmed milk for 1 h at room temperature, and then incubated with the respective primary antibody overnight at 4 °C. Anti-slow MyHC (Sigma, Cat. No. M8421), anti-fast MyHC (Sigma, Cat. No. M4276), PGC-1α (Affinity Biosciences, Cat. No. AF5395) and GAPDH (Absin, Cat. No. abs132004) were used. The membranes were washed six times, and subsequently incubated with secondary antibodies (CST) (1:2000 dilution in 5% milk/1 × TBST) for 1 h. Proteins were detected using an ECL reagent (Bio-Rad, Shanghai, China) on a Molecular Imager ChemiDoc XRS + System (Bio-Rad). The western blots were quantified using the ImageJ software (National Institutes of Health).

### Statistical analysis

Data were analysed using one-way ANOVA procedure of the SPSS software (version 21.0; SPSS Inc., Chicago, IL, USA). Duncan’s multiple range test was used to compare the differences between the groups with normally distributed data, while the data without a normal distribution were analysed using non-parametric analysis. Results are presented as mean ± standard error (SE). Differences were recognised as significant when *P* < 0.05, and a tendency was considered when 0.05 ≤ *P* < 0.10.

## Results

### Maternal organic Se supplementation increased the muscle area in LD muscle of weaning piglets

**Fig. 1:**
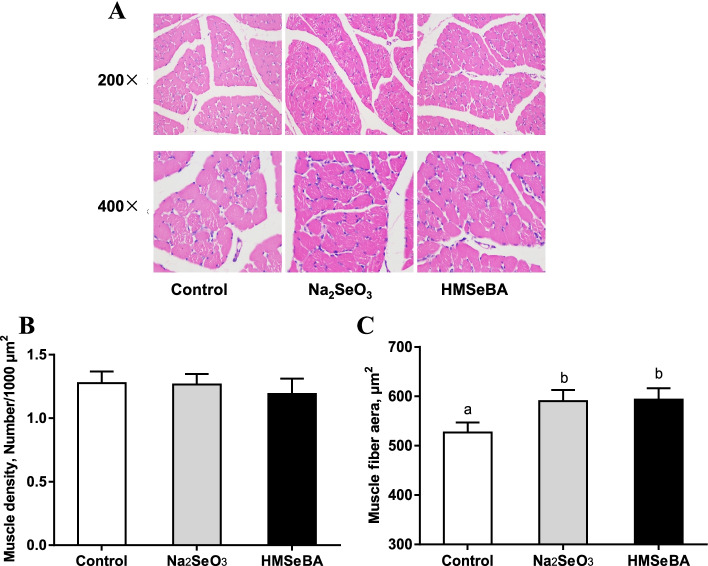
Maternal organic Se supplementation during gestation increased muscle fibre area in weaning piglets. **A** Muscle histology. **B** The muscle density in weaning piglets (*n* = 6). **C** Muscle fibre area in weaning piglets (*n* = 6). Data are presented as means ± SE. ^a,^^b^*P* < 0.05 between different superscripts

In this study, maternal organic Se supplementation increased the muscle area of the offspring, while there was no effect on muscle density (Fig. 1).

### Maternal organic Se supplementation increased the content of Se in LD muscle of newborn piglets

  Compared to that in the control and Na_2_SeO_3_ groups, maternal HMSeBA supplementation during gestation increased Se content in the LD muscle of newborn piglets (Table 3). Compared with the control group, maternal HMSeBA supplementation significantly reduced birth weight (*P* < 0.05) but had no effect on the weight or weight of LD as a percentage of body weight in newborn and weaned piglets. The body weights of the piglets were similar between the three groups at weaning (Table 3).

**Table 3:** Effect of maternal organic selenium supplementation during gestation on the weight and selenium content of offspring’s LD muscle

Item	Treatment			*P*-value
	**Control**	**Na**_**2**_**SeO**_**3**_	**HMseBA**	
Newborn piglets (*n* = 10)				
Birthweight, kg	1.57 ± 0.04^b^	1.49 ± 0.07^ab^	1.36 ± 0.04^a^	0.030
Weight of LD, g	13.49 ± 0.64	12.60 ± 0.94	11.59 ± 0.72	0.244
Weight of LD:BW, %	0.85 ± 0.02	0.84 ± 0.03	0.84 ± 0.03	0.825
Selenium content, mg/kg	0.068 ± 0.006^a^	0.051 ± 0.002^a^	0.139 ± 0.006^b^	0.000
Weaned piglets (*n* = 6)				
Body weight, kg	5.70 ± 0.31	5.80 ± 0.16	5.42 ± 0.12	0.221
Weight of LD, g	74.28 ± 7.21	69.59 ± 3.43	64.74 ± 2.12	0.391
Weight of LD:BW, %	1.29 ± 0.06	1.20 ± 0.03	1.20 ± 0.03	0.247

*LD* *Longissimus dorsi*, *BW* Body weight. Data were expressed as the mean ± SE

^a,b^*P* < 0.05 between different superscripts within the same line

### Maternal organic Se supplementation changed the expression of muscle development-related genes in offspring

**Fig. 2:**
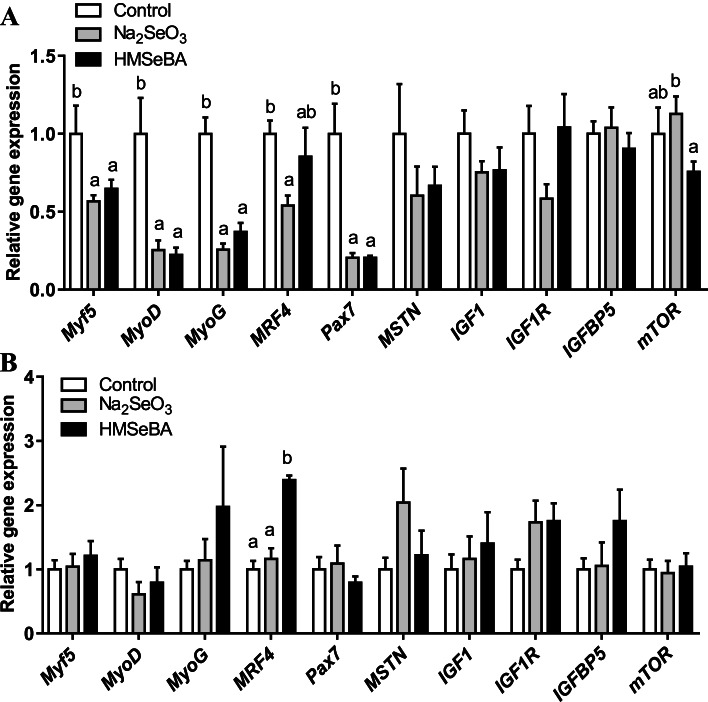
Maternal organic Se supplementation during gestation changed the expression of muscle development-related genes in offspring. **A** The expression of muscle development-related genes in newborn piglets (*n* = 10). **B** The expression of muscle development-related genes in weaned piglets (*n* = 6). *Myf5*, myogenic factor 5; *MyoD*, myogenic differentiation antigen; *MyoG*, myogenin; *MRF4*, myogenic regulatory factor 4; *Pax7*, paired box 7; *MSTN*, Myostatin; *IGF1*, insulin-like growth factor 1; *IGF 1R*, IGF receptor type 1; *IGFBP5*, insulin-like growth factor-binding protein-5; *mTOR*, mammalian target of rapamycin. Data are presented as means ± SE. ^a,b^*P* < 0.05 between different superscripts within the same gene

Compared to the control group, maternal HMSeBA and Na_2_SeO_3_ supplementation decreased the mRNA levels of *Myf5*, *MyoD*, *MyoG*, and *Pax7* (*P* < 0.05), whereas only maternal Na_2_SeO_3_ supplementation reduced the expression of *MRF4* in newborn piglets (*P* < 0.05) (Fig. 2A). Maternal organic Se supplementation during gestation decreased the expression of *mTOR* compared to that in the Na_2_SeO_3_ group (*P* < 0.05) (Fig. 2A). Moreover, in weaned piglets, maternal HMSeBA supplementation increased the expression of *MRF4* compared with that in the Na_2_SeO_3_ and control groups (*P* < 0.05) (Fig. 2B).

### Maternal organic Se supplementation during gestation changed muscle fibre type in offspring

**Fig. 3:**
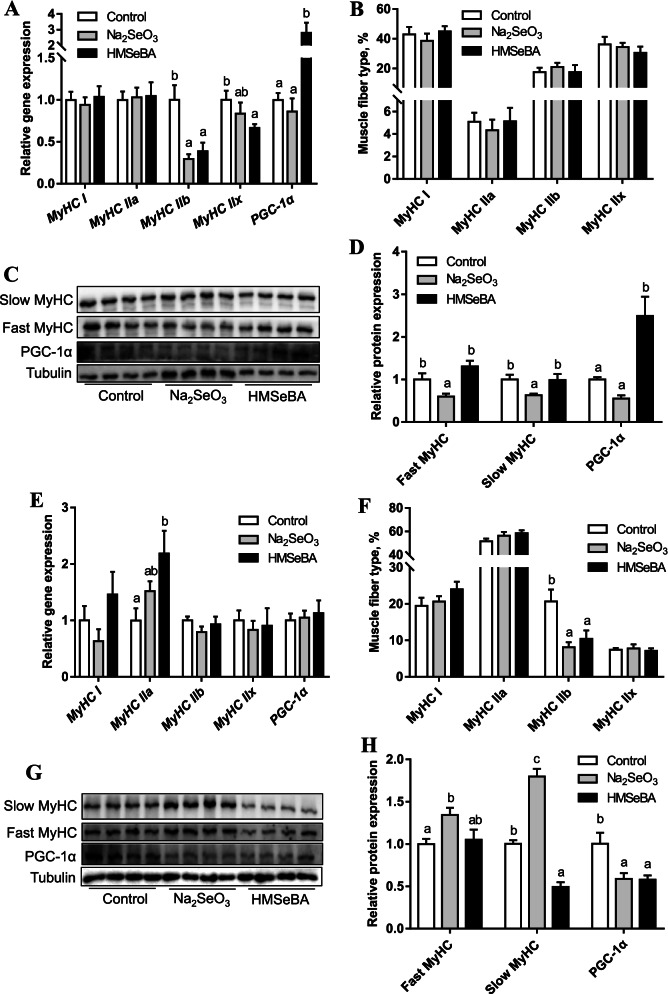
Effect of maternal HMSeBA supplementation during gestation on the expression of muscle fiber type-related genes in the LD muscle of offspring. **A** The expression of muscle fiber type-related genes in newborn piglets (*n* = 10). **B** The percentage of muscle fiber type in newborn piglets (*n* = 10). **C** The protein levels of slow MyHC, fast MyHC and PGC-1α in newborn piglets. **D** Quantification for proteins of newborn piglets. **E** The expression of muscle fiber type-related genes in weaned piglets (*n* = 6). **F** The percentage of muscle fiber type in weaned piglets (*n* = 6). **G** The expression of slow MyHC, fast MyHC and PGC-1α proteins in weaned piglets. **H** Quantification for proteins of weaned piglets. MyHC I, myosin heavy chain type1; MyHC IIa, myosin heavy chain type 2a; MyHC IIb, myosin heavy chain type 2b; MyHC IIx, myosin heavy chain type 2x; PGC-1a, peroxisome proliferator-activated receptor gamma coactivator-1 alpha; GAPDH, glyceraldehyde 3-phosphate dehydrogenase. Data are presented as means ± SE. ^a,b,c^*P* < 0.05 between different superscripts within the same gene or protein

The mRNA levels of *MyHC I, MyHC IIa, MyHC IIb*, and *MyHC IIx* in the LD muscle of newborn piglets were analysed. The results showed that, compared with the control group, maternal HMSeBA supplementation decreased the mRNA expression of *MyHC IIb* and *MyHC IIx* (*P* < 0.05) and increased the expression of *PGC-1α*, while maternal Na_2_SeO_3_ supplementation only decreased the mRNA expression of *MyHC IIb *(Fig. 3A). In addition, the percentage of MyHC IIb fibres was reduced in both the Na_2_SeO_3_ and HMSeBA groups compared with the control group (*P* < 0.05) (Fig. 3B). Additionally, the protein level of PGC-1α was increased in the HMSeBA group when compared with the control and Na_2_SeO_3_ groups (*P* < 0.05), whereas there was no difference in the protein expression of slow MyHC and fast MyHC (Fig. 3C, D).

In weaned piglets, maternal HMSeBA supplementation increased the mRNA level of *MyHC* *IIa* compared with that in the control group (*P* < 0.05) (Fig. 3E). However, maternal HMSeBA supplementation did not change the percentage of MyHC I, MyHC IIa, MyHC IIb, or MyHC IIx (Fig. 3F). Piglets from the HMSeBA group had lower slow MyHC and PGC-1α levels than piglets from the control group (*P* < 0.05), while piglets from the Na_2_SeO_3_ group had higher slow MyHC and fast MyHC and lower PGC-1α levels than piglets from the control group (Fig. 3G, H).

### Effects of maternal organic Se supplementation during gestation on the activities of metabolic enzymes in the LD muscle of the offspring

**Fig. 4:**
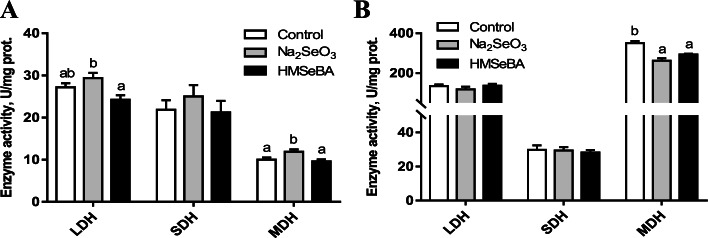
Effect of maternal HMSeBA supplementation during gestation on the activities of metabolic enzymes in piglets. **A** Metabolic enzyme activities in newborn piglets (*n* = 10). **B** Metabolic enzyme activities in weaned piglets (*n* = 6). LDH, lactate dehydrogenase; SDH, succinate dehydrogenase; MDH, malate dehydrogenase. Data are presented as means ± SE. ^a,b^*P* < 0.05 between different superscripts within the same enzyme

The activities of LDH and MDH in the LD muscle of newborn piglets were lower (*P* < 0.05) in the HMSeBA group than in the Na_2_SeO_3_ group (Fig. 4A). In addition, MDH activity in weaned piglets was lower (*P* < 0.05) in both the HMSeBA group and Na_2_SeO_3_ group than in the control group (Fig. 4B).

### Organic Se supplementation increased antioxidation indicators in LD muscle of offspring

Newborn piglets from the HMSeBA group had higher muscle GSH-Px activity and lower MDA content (*P* < 0.05) than newborn piglets from the Na_2_SeO_3_ and control groups and had higher T-SOD activity (*P* < 0.05) than newborn piglets from the control group (Table 4). Besides, the MDA content in the LD muscle of weaned piglets was lower (*P* < 0.05) in the HMSeBA group than in the control and Na_2_SeO_3_ groups (Table 4).

**Table 4:** Effect of maternal selenium supplementation during gestation on the oxidative status of offspring

Item	Treatment			*P*-value
	**Control**	**Na**_**2**_**SeO**_**3**_	**HMseBA**	
Newborn piglets (*n* = 10)				
MDA, nmol/mg prot	6.97 ± 0.29^b^	7.20 ± 0.70^b^	5.45 ± 0.21^a^	0.002
T-AOC, U/mg prot	0.38 ± 0.05	0.46 ± 0.05	0.44 ± 0.04	0.418
CAT, U/mg prot	12.74 ± 0.61	14.20 ± 0.87	12.73 ± 0.38	0.217
T-SOD, U/mg prot	421.76 ± 22.65^a^	462.08 ± 20.15^ab^	517.08 ± 19.74^b^	0.012
GSH-P_X_, U/mg prot	30.16 ± 5.31^a^	46.78 ± 7.41^a^	78.70 ± 13.60^b^	0.014
Weaned piglets (*n* = 6)				
MDA, nmol/mg prot	4.09 ± 0.20^b^	3.96 ± 0.44^b^	2.27 ± 0.35^a^	0.003
T-AOC, U/mg prot	0.46 ± 0.06	0.40 ± 0.01	1.12 ± 0.34	0.467
CAT, U/mg prot	12.38 ± 1.27	13.16 ± 0.85	12.12 ± 1.04	0.797
T-SOD, U/mg prot	350.18 ± 6.40	359.85 ± 15.60	375.37 ± 6.70	0.261
GSH-P_X_, U/mg prot	44.80 ± 5.19	41.68 ± 6.07	46.24 ± 3.73	0.805

*MDA* Malondialdehyde, *T-AOC* Total antioxidant capability, *CAT* Catalase, *T-SOD* Total superoxide dismutase, *GSH-P*_*X*_ Glutathione peroxidase. Data were expressed as the mean ± SE. ^a,b^*P* < 0.05 between different superscripts within the same line

### Maternal organic Se supplementation regulated the expression of selenoprotein genes in LD muscle of offspring

**Fig. 5:**
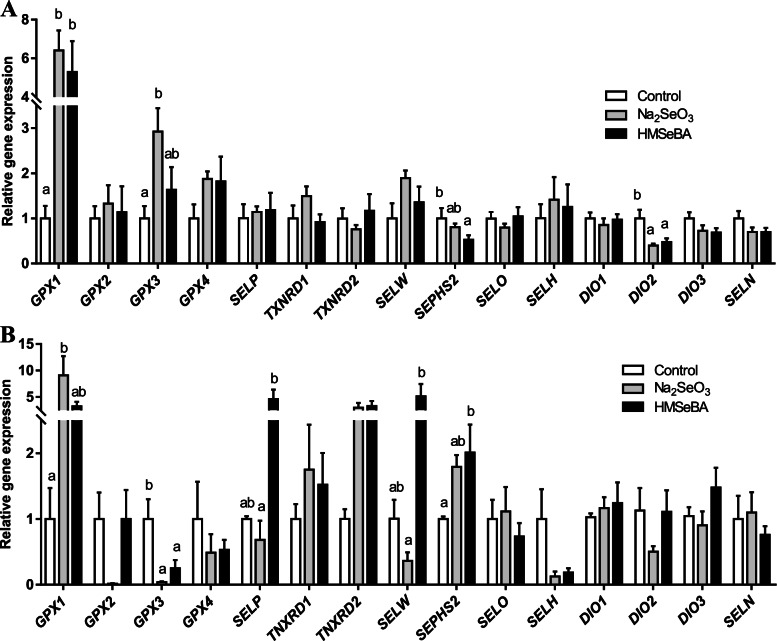
Effect of maternal HMSeBA supplementation during gestation on the expression of selenoprotein genes in LD muscle of offspring. **A** The expression of selenoprotein genes in LD muscle of newborn piglets (*n* = 10). **B** The expression of selenoprotein genes in LD muscle of weaned piglets (*n* = 6). *GPX*, Glutathione peroxidase; *SELP*, Selenoprotein P; *TXNRD*, Thioredoxin reductase; *SELW*, selenoprotein W; *SEPHS2*, Selenophosphate synthetase 2; *SELO*, Selenoprotein O; *SELH*, Selenoprotein H; *DIO*, Iodothyronine deiodinase; *SELN*, Selenoprotein N. Data are shown as means ± SE. ^a,b^*P* < 0.05 between different superscripts within the same gene

We then analysed the mRNA levels of selenoproteins in the LD muscle. Results showed that maternal HMSeBA supplementation during gestation increased the mRNA expression of *GPX1* and decreased the mRNA expression of *SEPHS2* and *DIO2* (*P* < 0.05) compared to the control group in the LD muscle of newborn piglets (Fig. 5A). Besides, the mRNA expression of *GPX1* and *GPX3* was higher (*P* < 0.05) and the mRNA expression of *DIO2* was lower (*P* < 0.05) in the newborn piglets of the Na_2_SeO_3_ group than in the control group (Fig. 5A).

In the LD muscle of weaned piglets, maternal HMSeBA supplementation increased the expression of *SEPHS2* (*P* < 0.05) while Na_2_SeO_3_ supplementation increased the expression of *GPX1* (*P* < 0.05) compared to the control group. Both HMSeBA supplementation and Na_2_SeO_3_ supplementation decreased the expression of *GPX3* (*P* < 0.05) compared to the control group (Fig. 5B). Compared with the Na_2_SeO_3_ group, maternal HMSeBA supplementation increased the expression of *SELP* and *SELW* (*P* < 0.05) (Fig. 5B).

## Discussion

Although there have been many studies on Se nutrition, there is little research on the effect of maternal Se nutrition on the development of offspring muscle fibres in swine models. It is well known that the muscle occupies an important position in animal production, and the foetal period is important for muscle development. Our findings revealed that maternal HMSeBA supplementation during gestation increased the muscle area. Diniz et al. [23] found that maternal organic Se supplementation during late gestation resulted in the upregulation of myosin and actin filament-associated genes in newborn calves, potentially allowing for optimal muscle function and contraction. Excessive oxidative stress may be a key factor in early foetal loss [24], whereas moderation of oxidative stress can promote muscle development during the embryonic period through the Wnt signalling pathway [25]. Wnt proteins are known to be involved in myogenesis as they can regulate the expression of *Pax3*, *Pax7*, and *MRF* genes [26]. Our results showed that compared with control, maternal organic Se supplementation during gestation significantly decreased the gene expression of *myf5*, *MyoD*, *MyoG*, and *Pax7* in the LD muscle and the body weight of newborn piglets. However, at weaning, the expression of *MRF4* mRNA was significantly increased compared to the control and Na_2_SeO_3_ groups, while the body weight and LD muscle weight were similar among the three groups. These data suggest that piglets in the HMSeBA group experienced catch-up growth [27] during the newborn period and will have better muscle development potential because of the higher *MRF4* expression [28].

Selenoprotein W (SELW), without a known biological function [29], is the most widely distributed selenoprotein in muscles under normal conditions [30]. Therefore, SELW may be involved in muscle metabolism. Loflin et al. [31] showed that SELW is involved in muscle growth and differentiation. Li et al. [32] found that increased expression of the *SELW* gene was associated with enhanced water-holding capacity in meat. In our study, we showed an increase in *SELW* gene expression in weaned piglets in the HMSeBA group compared to the Na_2_SeO_3_ group, which suggests that piglets in the HMSeBA group might have better muscle development and meat quality in the future. Further studies with growing pigs are required to confirm this.

The perinatal period is critical for muscle development in piglets [16]. If muscle development is restricted during this period, muscle growth is affected, resulting in permanent damage [33]. Lefaucheur et al. [34] found that undernutrition during the first postnatal week could decrease hypertrophy of the future fast-twitch glycolytic fibres, delay contractile and metabolic maturation in later maturation processes, and increase the percentage of MyHC I-containing fibres in the psoas muscle. The activities of SDH, MDH, and LDH are considered indicators of muscle oxidation and glycolysis. Several reports have shown that the activity of SDH and MDH is higher in oxidised fibres than in glycolytic fibres, while the activity of LDH in glycolytic fibres is higher than that in oxidised fibres [21, 35, 36]. In the present study, our results indicated that more oxidised muscle fibres were transformed into glycolytic muscle fibres in the HMSeBA group during the period between birth and weaning. In addition to the change in PGC-1α expression, adequate Se leads to higher feed intake [37] and improved antioxidant status [38] which may be another reason for this phenomenon.

It is well known that maternal nutrition during pregnancy has a profound impact on foetal development. Se can be added as an antioxidant to sow diets during pregnancy and lactation [39]. Dietary selenium can be used to synthesise selenoprotein P in the liver, which can then be transferred to the foetus through the cord blood, placenta [40], colostrum and milk [41], and other transport systems. The Se in the foetus is then deposited in different tissues, and supplied to some Se-containing proteins according to a hierarchy in selenoprotein expression which play different roles in different tissues [42]. Chao et al. [3] showed that HMSeBA supplementation increased Se content in the muscle compared to Na_2_SeO_3_ supplementation. In addition, Se concentrations in neonatal pigs from sows fed yeast Se was higher than those fed Na_2_SeO_3_ [43]. Our study also found that maternal HMSeBA supplementation increased Se content in the LD muscle of newborn piglets compared to the control and Na_2_SeO_3_ groups. The higher efficiency of organic Se in absorption, tissue accumulation, and antioxidant bioavailability [3, 4] may be the reason for this.

Newborn piglets suffer from severe oxidative stress at birth owing to their incomplete antioxidant system [44]. Therefore, the development and growth process of the foetus is easily affected by oxidative stress, and this negative effect may extend to later stages in life. In the current study, we found that the activities of muscle GSH-Px and T-SOD in newborn piglets were significantly increased, while MDA content was decreased in the HMSeBA group compared to the control group and Na_2_SeO_3_ group. This result suggests that maternal HMSeBA supplementation during gestation improves the antioxidant capacity of the foetus. Furthermore, TXNRD2 expression was higher in the HMSeBA group than in the control group. These results were similar to the results of Zhan et al. who found that maternal selenomethionine supplementation during gestation and lactation improved the antioxidant status in muscle as compared to the Na_2_SeO_3_ supplementation [7]. These results indicate that maternal supplementation of organic selenium during pregnancy can improve not only the redox status in the LD muscle of newborn piglets but also the redox status in weaned piglets. The half-life of Se in muscles is 12 d [45]. Sows fed organic Se had a greater transfer efficiency of Se to the neonate, colostrum, milk, weaned piglets, and sow tissues than sows fed inorganic Se. Our previous study also found that SELP content in milk on day 7 of lactation in the HMSeBA group was higher than that on day 0 of lactation [8]. These data suggest that piglets from the organic Se group take up more Se from sows through maternal milk, and Se can last longer in organic form. This may be one of the reasons for the improvement in the antioxidant status in the muscle of weaned piglets.

## Conclusion

The present study showed that maternal HMSeBA supplementation during pregnancy increased muscle Se deposition in newborn piglets and improved the antioxidative capacity and development of offspring muscle. It is very interesting that there are significant changes in muscle fibres during birth and weaning. Current results indicate maternal organic Se supplementation during gestation may be beneficial for muscle development in the offspring.

## Data Availability

All data generated or analyzed during this study are included.

## Abbreviations

HMSeBA: 2-Hydroxy-4-methylselenobutanoic acid
Na2SeO3: Sodium selenite
LD: *Longissimus dorsi*
LDH: Lactate dehydrogenase
SDH: Succinate dehydrogenase
MDH: Malate dehydrogenase
T-SOD: Total superoxide dismutase
GSH-Px: Glutathione peroxidase
CAT: Catalase
T-AOC: Total antioxidant capability
MDA: Malondialdehyde
Myf5: Myogenic factor 5
MyoD: Myogenic differentiation antigen
MyoG: Myogenin
MRF4: Myogenic regulatory factor 4
Pax 7: Paired box 7
MSTN: Myostatin
IGF1: Insulin-like growth factor 1
IGF1R: IGF receptor type 1
IGFBP5: Insulin-like growth factor-binding protein-5
mTOR: Mechanistic target of rapamycin
MyHC 1: Myosin heavy chain type1
MyHC IIa: Myosin heavy chain type 2a
MyHC IIb: Myosin heavy chain type 2b
MyHC IIx: Myosin heavy chain type 2x
PGC-1a: Peroxisome proliferator-activated receptor gamma coactivator-1 alpha
GAPDH: Glyceraldehyde 3-phosphate dehydrogenase
GPX1-4: Glutathione peroxidase 1–4
SELP: Selenoprotein P
TXNRD1-2: Thioredoxin reductase 1 -2
SELW: Selenoprotein W
SEPHS2: Selenophosphate synthetase 2
SELO: Selenoprotein O
SELH: Selenoprotein H
DIO1-3: Iodothyronine deiodinase 1–3
SELN: Selenoprotein N
